# Zebrafish as a model organism for neurodegenerative disease

**DOI:** 10.3389/fnmol.2022.940484

**Published:** 2022-10-13

**Authors:** Kelda Chia, Anna Klingseisen, Dirk Sieger, Josef Priller

**Affiliations:** ^1^Centre for Clinical Brain Sciences, University of Edinburgh, Edinburgh, United Kingdom; ^2^United Kingdom Dementia Research Institute at University of Edinburgh, Edinburgh, United Kingdom; ^3^Centre for Discovery Brain Sciences, University of Edinburgh, Edinburgh, United Kingdom; ^4^Department of Psychiatry and Psychotherapy, School of Medicine, Technical University of Munich, Munich, Germany; ^5^Neuropsychiatry and Laboratory of Molecular Psychiatry, Charité - Universitätsmedizin Berlin, DZNE, Berlin, Germany; ^6^Department of Psychological Medicine, Institute of Psychiatry, Psychology and Neuroscience, King’s College London, London, United Kingdom

**Keywords:** zebrafish, Alzheimer’s disease, multiple sclerosis, Parkinson’s disease, amyotrophic lateral sclerosis (ALS), Huntington’s disease (HD)

## Abstract

The zebrafish is increasingly recognized as a model organism for translational research into human neuropathology. The zebrafish brain exhibits fundamental resemblance with human neuroanatomical and neurochemical pathways, and hallmarks of human brain pathology such as protein aggregation, neuronal degeneration and activation of glial cells, for example, can be modeled and recapitulated in the fish central nervous system. Genetic manipulation, imaging, and drug screening are areas where zebrafish excel with the ease of introducing mutations and transgenes, the expression of fluorescent markers that can be detected *in vivo* in the transparent larval stages overtime, and simple treatment of large numbers of fish larvae at once followed by automated screening and imaging. In this review, we summarize how zebrafish have successfully been employed to model human neurodegenerative diseases such as Parkinson’s disease, Alzheimer’s disease, amyotrophic lateral sclerosis, and Huntington’s disease. We discuss advantages and disadvantages of choosing zebrafish as a model for these neurodegenerative conditions.

## Introduction

The zebrafish (Danio rerio) is a tropical freshwater teleost fish that is part of the Cyprinidae family that was introduced into biological research by George Streisinger in the 1970s. He selected zebrafish for three reasons: the generation of large numbers of embryos accessible from the one-cell-stage, the optical clarity of the developing fish, and a diploid genome allowing for genetic studies. Several large scale forward genetic screens by Nobel Prize laureate Christiane Nüsslein-Volhard and others firmly established the zebrafish as an ideal model system for developmental biology (Mullins et al., 1994; Haffter et al., 1996; Amsterdam et al., 1999; Draper et al., 2004). Today, the zebrafish is gaining traction for the study of human diseases. Here, we focus on the use and usefulness of the zebrafish model to study human neurodegenerative diseases such as Parkinson’s disease (PD), Alzheimer’s disease (AD), amyotrophic lateral sclerosis/Lou Gehrig’s disease (ALS), and Huntington’s disease (HD).

**FIGURE 1:**
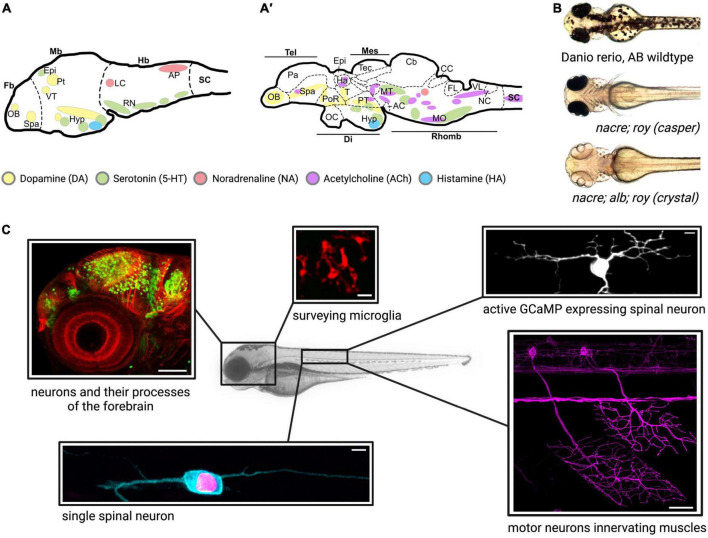
**(A,A′)** Schematic illustration of the monoaminergic localities and anatomy of the zebrafish brain. The monoaminergic system is well developed in the zebrafish, with brain specific regions of neuronal clusters for the different neurotransmitters, dopamine (yellow), noradrenaline (red), serotonin (green), acetylcholine (purple), and histamine (blue). **(A)** The brain is developed and CNS fully functional in the larval zebrafish from as early as 3 dpf. **(A′)** The schematic lateral view of the adult zebrafish brain shows a well-defined and complex architecture. Schematic representation and annotations have been adapted from **(A)** (Alunni et al., 2013) and **(B)** Wullimann et al. (2011), Parker et al. (2013). AC, ansulate commissure; AP, area postrema; Cb, cerebellum; CC, crista cerebellaris; Di, diencephalon; Epi, epiphysis; Fb, forebrain; FL, facial lobe; Ha, habenula; Hb, hindbrain; Hyp, hypothalamus; LC, locus coeruleus; Mb, midbrain; Mes, mesencephalon; MO, medulla oblongata; MT, mesencephalic tegmentum; NC, nucleus commisuralis; OB, olfactory bulb; OC, optic chiasma; Pa, pallium; PoR, preoptic region; Pt, pretectum; PT, posterior tuberculum; Rhomb, rhombencephalon; RN, raphé nuclei; SC, spinal cord; Spa, subpallium; T, thalamus; Tec, tectum; Tel, telencephalon; VT, ventral thalamus; VL, vagal lobe. **(B)** The zebrafish is optically transparent during the first week of development. Lines for mutations in genes controlling pigment cell formation (*nacre*^w2/w2^, *roy*^a9/a9^) as well as melanin production (*alb*^b4/b4^) have been generated allowing for non-invasive *in vivo* imaging. Images in **(B)** have been taken and adapted from Antinucci and Hindges (2016) gp. **(C)** Examples of neuronal cell types imaged in zebrafish larvae. Neurons and processes in the forebrain [Dlx: GFP (green), tubulin (red) scale bar 100 μm]. Lateral view of a zebrafish embryo: *Monica Folgueira and Steve Wilson, Wellcome collection*, microglia (mpeg:mCherry) scale bar 10 μm, active spinal neuron (GCaMP7s) scale bar 5 μm, motor neurons (nefma:Kalt4UAS Scarlett) scale bar 20 μm, single spinal neuron [mTFP1(cyan), H2a-mCherry (red)], scale bar 5 μm.

Zebrafish development is conveniently fast. Zebrafish are sexually mature by 12 weeks of age and can produce several hundred embryos each week (Lawrence et al., 2012) which develop ex utero, allowing for experimental manipulation as well as observation from the one-cell stage. All major organs and the central nervous system (CNS) are fully functional by 72 h post-fertilization (hpf) (Fulwiler and Gilbert, 1991; Kimmel et al., 1995; Blader and Strähle, 2000; Schmidt et al., 2013). The zebrafish brain shows structural and functional similarities in its anatomy with the mammalian brain, as well as corresponding neural circuitry including most major neurochemical signal transduction pathways (Fulwiler and Gilbert, 1991; Blader and Strähle, 2000; Postlethwait et al., 2000; Howe et al., 2013; Schmidt et al., 2013; Figures 1A,A′). The neurotransmitter system is conserved in the zebrafish from early in development, sharing clear similarities with the mammalian systems such as dopaminergic cell clusters in the olfactory bulb and hypothalamus, and producing neurotransmitters such as dopamine (DA), serotonin (5-HT), acetylcholine (ACh), histamine (HA), glutamate and GABA (Kaslin and Panula, 2001; Panula et al., 2006; Wasel and Freeman, 2020).

Genetically, the zebrafish shares a high level of conservation with humans. A comprehensive sequencing study demonstrated that zebrafish share at least one ortholog with over 70% of all human genes (Howe et al., 2013) including many of the risk genes identified in various human neurodegenerative diseases. Among these are genes implicated in the pathology of PD (*SNCA*, *PINK1*, *LRRK2*, and *Parkin*), familial AD (*PSEN1* and *PSEN2*), ALS (*SOD1*, *TARDBP*, *C9orf72*, and *FUS*), and HD (*Huntingtin*).

One of the major advantages of the zebrafish is their optical transparency throughout development (Figure 1B). This allows researchers to perform high resolution live *in vivo* imaging of the entire CNS (Figure 1C) without the need for small invasive optical windows commonly used in traditional mammalian research models (Godinho, 2011; Cong et al., 2017). The uncomplicated expression of transgenes with the binary UAS/Gal4 system allows for labeling of cells and structures as well as expression of human genes in all, specific or single neurons, for example (Kawakami et al., 2016). Human genes carrying disease-causing mutations can be easily expressed using this method to assess their impact on neuronal health. Additionally, advances in the CRISPR/Cas9 technology have greatly fine-tuned the tools of gene editing to introduce mutations and transgenes into the fish genome (Sager et al., 2010; Kimura et al., 2014; Liu et al., 2017; Rissone et al., 2020). Importantly, acute injection of highly active synthetic RNA Oligo CRISPR guide RNAs drastically speeds up the process as phenotypes can be assessed in the F0 generation and later confirmed by stable mutants (Keatinge et al., 2021). Injection of mRNA or DNA plasmids into the one-cell stage provides an uncomplicated and quick method to transiently express any gene/protein of interest or introduce new DNA into the embryo’s genome. State of the art imaging allows researchers to observe the migration of neuronal precursors, the movements of microglia, or neuronal cell death for example, in the intact animal and in real time (Formella et al., 2018). A single motor neuron can be labeled and followed during outgrowth, death, before and after injury, and in disease conditions (Kabashi et al., 2011; Zelenchuk and Brusés, 2011; Asakawa et al., 2021). The development of the fluorescent Ca2+ reporter GCaMP in the mid 1990s added another major tool for neurobiological studies by making neuronal activity imageable, and GCaMP was quickly adapted in zebrafish research (Dreosti and Lagnado, 2011; Akerboom et al., 2012; Kettunen, 2012). Neuronal activity of a single or cluster of neurons or indeed whole brain activity can now be monitored at rest or in the freely swimming larval fish (Ahrens et al., 2013; Bianco and Engert, 2015; Bergmann et al., 2018). Compared to electrophysiological recordings, optical monitoring of neuronal function allows for unprecedented spatial resolution as the fish offers optical access to the entire CNS to investigate both abnormal single cell as well as population behavior. Recently, another tool for understanding brain-wide neuronal dynamics was added by tracing a map from over 2,000 labeled neurons of the underlying circuit architecture of the larval zebrafish brain, building an interactive atlas at cellular resolution (Kunst et al., 2019). With the arrival of optogenetics, the activity of specific neural cells can now not only be monitored but also induced or silenced to study their downstream influence (Boyden, 2011; Wyart and Bene, 2011). Optogenetics can also modulate the multimerization status of a protein *in vivo* through external light illumination and thus be used to examine the dynamics and consequences of aggregate formation, a hallmark of many neurodegenerative diseases. This technique has for example been used to investigate TDP-34 aggregation in zebrafish motor neurons (Asakawa et al., 2020). The most recent development of a family of fluorescent biosensors called genetically encoded death indicators (GEDI) keep zebrafish at the forefront of neurodegenerative research. GEDIs detect a stage where a neuron is irreversibly committed to degeneration providing an earlier and more acute demarcation of the moment of death in a degenerating neuron than previously possible. Importantly, this is possible *in vivo* and in an un-anaesthetized animal (Linsley et al., 2021).

Despite all these advantages, embryonic fish and the larval stages are not always an ideal model for human neurodegenerative diseases with their adult onset, or even old age. As in mouse models, zebrafish may also not show the same pathological consequences upon gene deletion or mutation as in humans.

Here, we summarize how the zebrafish model has contributed to research into the pathogenesis of four human neurodegenerative diseases (PD, AD, ALS, and HD) and discuss on the background of recent advances in gene editing and manipulation of neuronal activity, how zebrafish can be a powerful model to investigate human neurodegenerative diseases.

## Parkinson’s disease

Parkinson’s disease (PD) is one of the most common neurodegenerative diseases with an annual estimated global incidence rate of between 5 and 35 per 100,000 individuals. PD is characterized by the combined loss of dopaminergic neurons in the substantia nigra, and intracellular α-synuclein accumulation in neurons of various brain regions (Lewy pathology) (Poewe et al., 2017). While PD has traditionally been perceived as a movement disorder, several non-motor- symptoms including autonomic dysfunction, cognitive deficits, as well as mood disorders are now recognized (Poewe et al., 2017). In the pathogenesis of PD, age is the most important contributing factor. While onset is generally rare before the age of 50, incidence rates increase with each increasing decade. Furthermore, life expectancy has been shown to significantly decline as PD progresses, with a mortality rate twice as high as the average within 10 years of disease onset (Pinter et al., 2015). As with most neurodegenerative diseases, the majority of PD cases are sporadic, with familial PD only accounting for around 5–10% of all cases. Genetically, mutations in synuclein α, P-TEN induced kinase 1 (*PINK1*), Leucine Rich Repeat Kinase 2 (*LRRK2*) and the E3 Ubiquitin Protein Ligase *Parkin*. Environmental factors such as frequent exposures to harmful chemicals such as pesticides or previous brain contusions in individuals also play a substantial role in developing PD (Bretaud et al., 2004). Caffeine and nicotine intake, however, have been associated with a decreased risk of PD. While current treatments have effectively increased quality of life and life expectancy in patients, PD remains incurable.

### Zebrafish models of Parkinson’s disease

Of the plethora of different neurodegenerative diseases and movement disorders, PD is by far one of the most established in the zebrafish model and its promise as a PD animal model for the development of therapeutics has been highlighted in a recent review (Razali et al., 2021). High conservation of PD-related genes and sensitivity to PD risk-related drugs in zebrafish have allowed for the generation of various genetic and transgenic, as well as chemically induced, models of PD (Tables 1, 2). Despite the absence of dopaminergic neurons in the zebrafish midbrain, a functional homolog of the mammalian substantia nigra is the diencephalic dopaminergic cluster within the posterior tuberculum (Rink and Wullimann, 2001), and the serotonergic and histaminergic systems present in the zebrafish show high resemblance to the mammalian system (Kaslin and Panula, 2001; Wang et al., 2006). While unable to recapitulate the full spectrum of the disease, the zebrafish model may be particularly useful to study PD-related hypokinetic syndromes. As will be presented below, phenotypic hallmarks mimicking bradykinesia in PD patients can be achieved in the zebrafish following induced dopaminergic cell aberrations.

**TABLE 1:** Genetic zebrafish models of PD.

Study	Method(s)	DA neuron loss	Other pathologies	Motor deficits	Other phenotypes
Anichtchik et al., 2008	PINK1 MO knockdown	Yes	ROS accumulation	Yes – Impaired TEER	Morphological deformities. Increased mortality.
Bretaud et al., 2007	DJ-1 MO knockdown	No	Increased sensitivity to oxidative stress. Increased sensitivity to proteasome inhibition. Upregulation of apoptosis regulator genes	Not reported	
Fett et al., 2010	Parkin MO knockdown	No	Increased susceptibility to proteotoxic stress	Not reported	
Flinn et al., 2009	Parkin MO knockdown	Yes	Reduced mitochondrial activity. Increased susceptibility to MPP+	No	
Flinn et al., 2014	TILLING-mediated PINK1 knockout	Yes	Mitochondrial dysfunction. Increased microglial numbers and activation.	Not reported	
Lulla et al., 2016	γ1-synuclein overexpression	Not reported	Synuclein aggregation	Not reported	Morphological deformities. Increased mortality.
Milanese et al., 2012	β-, and γ1-synuclein MO knockdown	Yes		Yes – Reduced spontaneous swim activity	
Prabhudesai et al., 2012	Human α-synuclein overexpression	gross neuronal apoptosis	Synuclein aggregation	Not reported	Developmental deformities. Embryonic lethality (100%).
Prabhudesai et al., 2016	LRRK2 MO knockdown	Yes	Synuclein aggregation	Not reported	Morphological deformities.
Priyadarshini et al., 2013	PINK1 MO knockdown, Microarray analysis	Not reported	Significant alteration of 177 genes. Increased ROS levels	Not reported	Reduced heart rate. Increased erythropoiesis.
Ren et al., 2011	ΔWD40-LRRK2 MO knockdown	No		No	
Sallinen et al., 2010	PINK1 MO knockdown	Yes	Increased vulnerability to MPTP exposure	Yes (post-MPTP treatment) – Reduced swim activity	
Sheng et al., 2010	LRRK2 MO knockdown	Yes		Not reported	Morphological deformities. Embryonic lethality (∼90%).
Sheng et al., 2010	ΔWD40-LRRK2 MO knockdown	Yes	Reduction and disorganization of axon tracts	Yes – Reduced swim activity	
Suzzi et al., 2021	LRRK2 CRISPR/Cas9-mediated knockout	No – But general increase in apoptosis	Reduced mitosis in larval brains. Impaired neuronal regeneration in adult brains.	Yes – Reduced spontaneous swim activity	
Xi et al., 2010	PINK1 MO knockdown	No – But disrupted patterning of the diencephalc DA neurons		Yes – Deficient TEER; reduced swim distance and speed; increased turning angle; preference to periphery of observation dish; difficulties in balancing	Increased mortality.
Zhao et al., 2012	FBXO7 MO knockdown	Yes		Yes – Reduced swim velocity	Morphological deformities. Increased mortality.

**TABLE 2:** Chemical zebrafish models of PD.

Study	Method(s)	DA neuron loss	Other pathologies	Motor deficits	Other phenotypes
Babu et al., 2016	MPTP	Not reported		Yes – Reduced swim movement and distance; reduced number of crosses; increased number of freezing bouts and duration	
Benvenutti et al., 2018	6-OHDA	Not reported		Yes – Reduced swim distance, speed, and maximum acceleration; increased absolute turn angle; increased immobility time	Reduced head and total length.
Bortolotto et al., 2014	Paraquat	Not reported		Yes – Reduced swim distance and velocity; impaired motor coordination; reduced line crossings	
Bretaud et al., 2004	MPTP	Yes		Yes – Reduced swimming activity	In adults – Respiratory dysfunction and darkened pigmentation.
Bretaud et al., 2004	Paraquat	No		No	
Bretaud et al., 2004	Rotenone	No		No	
Byrnes et al., 2018	Rotenone	Not reported – But induced brain death phenotype	Reduced skeletal muscle mitochondrial membrane potential	Yes – Reduced responsiveness	Developmental deformities.
Cronin and Grealy, 2017	6-OHDA	Yes		Yes – Reduced locomotor activity	Morphological deformities.
Feng et al., 2014	6-OHDA	Not reported		Yes – Reduced swim distance; increased time spent in the bottom zone of tank	
Khotimah et al., 2015	Rotenone	Yes	Synuclein aggregation. Increased apoptosis and caspase expression. Reduced BDNF expression.	Yes – Reduced swim motility	
Lam et al., 2005	MPTP	Yes		Yes – Impaired TEER; reduced swim distance and velocity	
Martel et al., 2015	Rotenone	Yes	Increased susceptibility to oxidative stress	Yes – Reduced tank midline crossing	
McKinley et al., 2005	MPTP	Yes		Not reported	
Melo et al., 2015	Rotenone	Not reported		Yes – Impaired TEER; impaired balance; mydriasis; tremors; paralysis; erratic swimming	Developmental deficits. Morphological deformities.
Nellore and Nandita, 2015	Paraquat	reduced dopamine levels; increased cell apoptosis		Yes – Reduced swim distance; impaired tail coiling	Developmental deformities. Increased mortality.
Nunes et al., 2017	Paraquat	Not reported	Reduced mitochondrial viability	Yes – Increased time spent in the top zone of the tank;	Increased aggressiveness.
Sallinen et al., 2009	MPTP	Yes		Yes – Reduced swim distance and speed	
Vijayanathan et al., 2017	6-OHDA	Yes		Yes – Reduced swim distance and speed	
Wang et al., 2016	Paraquat	Not reported – But increased cell apoptosis	Increased susceptibility to oxidative stress. Increased migration and activation of macrophages.	Not reported	Increased mortality.
Wang et al., 2017	Rotenone	Yes		Yes – Reduced swim duration and distance at fast speed; increased time spent in the light vs. dark	Olfactory dysfunction.
Wang et al., 2018	Paraquat	Not reported	Reduced maximum respiration rate. Increased susceptibility to oxidative stress.	Yes – increased swim distance, duration, and velocity	Accelerated hatching.
Wen et al., 2008	MPTP	Yes		Not reported	
Zhang et al., 2015	6-OHDA	Yes		Yes – Reduced swim distance	
Zhang et al., 2016	6-OHDA	Yes		Yes – Reduced swim distance	

#### Synucleins

One of the most recognizable PD-associated genes in humans is the *SNCA* gene encoding α-synuclein. α-synuclein mutations are linked to early onset familial PD, and α-synuclein aggregation in Lewy bodies is associated with sporadic cases of PD (Meade et al., 2019). Although the physiological function of α-synuclein is still unclear, it is believed to be responsible for regulating the synaptic transmission process (Marques and Outeiro, 2012; Burré, 2015). However, in pathological conditions, misfolded α-synuclein proteins form aggregates, which contribute to the formation of Lewy bodies (LBs) in neurons (Mahul-Mellier et al., 2020).

Synucleins are a family of neuronal proteins consisting of α-, β-, and γ-synuclein. While the zebrafish does not express an ortholog of the human α-synuclein gene, the β-, and γ-synucleins can be found in the zebrafish genome and are expressed in three isoforms. *sncb*, *sncg1*, and *sncg2* that encode for β-, γ 1-, and γ2-synucleins, respectively, which seem to compensate for the absence of α-synuclein. Importantly, zebrafish and human synucleins share high sequence similarity, with the zebrafish γ1-synuclein functionally most similar to human α-synuclein (Sun and Gilter, 2008). Milanese et al. demonstrated that knockdown of both zebrafish β- and γ1-synucleins resulted in severe impairment of the differentiation of dopaminergic neurons and the development of the dopamine system (Milanese et al., 2012). This in turn led to hypokinetic motor behavior such as reduced spontaneous swim activity, indicating that zebrafish β- and γ1-synuclein proteins are required for movement regulation and dopamine homeostasis (Toni and Cioni, 2015; Vaz et al., 2018; Robea et al., 2020). Both motor and dopaminergic deficits could be rescued by exogenous expression of human α-synuclein (Milanese et al., 2012). In line with the ability of human α-synuclein to revert phenotypes caused by the loss of β- and γ1-synucleins in the zebrafish, Prabhudesai et al. (2012) generated a model to overexpress human α-synuclein in the zebrafish embryo. While evidence for LBs formation typical in human PD pathology remains to be determined, accumulation of α-synuclein induced significant neurotoxicity in the developing embryo leading to widespread neuronal cell death, morphological deformities, as well as rapid mortality. Further studies found that over-expression and aggregation of human α-synuclein protein in zebrafish larvae led to reduced mitochondrial activity and increased presence of reactive oxygen species (ROS), which led to neuronal apoptosis and cell death (O’Donnell et al., 2014; Robea et al., 2020).

#### P-TEN induced kinase 1

The PTEN-induced kinase 1 (*PINK1*) gene that encodes for PINK1 protein plays a critical role in regulating oxidative stress and protecting neurons against mitochondrial dysfunctions (Gonçalves and Morais, 2021). Mutations in the *PINK1* gene leading to reduced PINK1 protein translation is a major cause to autosomal recessive early onset PD. In a pioneering study investigating loss-of-function of PINK1 in zebrafish, Anichtchik et al. observed significant morphological abnormalities and phenotypes including axonal tract deformations, impaired escape responses, and increased mortality following PINK1 knockdown by morpholino (MO) injection. Importantly, Acridine Orange assays and oxidative probes demonstrated increased neuronal cell death and accumulation of ROS, reflecting the increased susceptibility of neurons to oxidative insults and cell death following damage of cellular mitochondria (Anichtchik et al., 2008). This was a significant observation as previous investigations of PINK1 knockout (KO) mice did not reveal dopaminergic cell loss (Kitada et al., 2007). In slight discrepancy, Xi et al. observed severely disorganized patterning of the dopaminergic neurons but no increased cell loss in PINK1 morphants. The authors did, however, observe loss of balance, retarded responses to tactile stimuli, as well as significantly reduced swim distance and speed following PINK1 knockdown (Xi et al., 2010). Another study using morpholino-mediated knockdown of PINK1 showed a decrease in TH (tyrosine hydroxylase)-positive dopaminergic neurons in specific regions within the diencephalon, which was exacerbated when PINK1 morphants were exposed to the neurotoxin 1-methyl-4-phenyl-1,2,3,6-tetrahydropyridine (MPTP) that also caused motor deficits (Sallinen et al., 2010). To investigate the functional role of PINK1 on downstream genes and pathways, Priyadarshini et al. (2013) carried out a microarray analysis and showed that expression of 177 genes was significantly altered following PINK1 depletion. Downstream pathways affected included processes of mitochondrial function, as well as TGF-β signaling, with the most impacted pathway found to be hypoxia-induced factor (HIF) signaling. ROS expression levels were significantly increased in the PINK1 morphants providing further evidence that PINK1 loss-of-function leads to increased oxidative stress and an inflammatory environment (Priyadarshini et al., 2013). Subsequently, stable PINK1 null (*pink1*^–/–^) mutant zebrafish were generated that showed persistent loss of dopaminergic neurons in the absence of severe morphological abnormalities (Flinn et al., 2014). In addition to mitochondrial impairment and dysfunction following PINK1 KO, Flinn et al. observed significant microgliosis in the *pink1*^–/–^ mutants. This was a significant observation as it provided the first insight on the impact on immune cells in the context of a zebrafish PD model.

#### Leucine rich repeat kinase 2

The most common cause of autosomal late onset PD, mutations in leucine-rich repeat kinase 2 (*LRRK2*) that encodes the LRRK2 protein account for 5–13% of familial PD and 1–5% of sporadic PD (Drolet et al., 2011). Under physiological conditions, LRRK2 regulates kinase activity important for controlled protein degradation and immune response (Li et al., 2014). In the context of PD, LRRK2 function has been implicated in pathologic α-synuclein aggregation and the internalization and attenuation of its pathological propagation by vigilant microglia (Rui et al., 2018). Furthermore, PD patients with *LRRK2* mutations show dysregulated kinase activity leading to increased ROS production and proinflammatory responses resulting ultimately in dopaminergic cell death (Li et al., 2014). Initial studies modeling LRRK2 deficiency in the zebrafish using MO knockdowns reported slightly contradicting findings on dopaminergic neuronal loss as well as locomotor deficits (Sheng et al., 2010; Ren et al., 2011; Prabhudesai et al., 2016). However, the recent generation of a genetic zebrafish *LRRK2* deletion by CRISPR/Cas9 confirmed the finding of increased apoptotic cell death within the brain parenchyma, albeit not specific to neurons (Suzzi et al., 2017). Furthermore, *LRRK2* mutants showed reduced mitosis and proliferation leading to impaired neurogenesis and neuronal regeneration, as well as locomotor deficits and impaired swimming patterns similar to PD-related bradykinesia (Suzzi et al., 2017).

#### Parkin

A leading cause of autosomal recessive early onset PD arises from mutations in the E3 ubiquitin protein ligase Parkin, which regulates the ubiquitination of proteins required for dopaminergic cell survival (Pickrell and Youle, 2015) as well as mitochondrial processes (Kamienieva et al., 2021). Mitochondrial and metabolic dysfunction following the loss-of-function of Parkin has been shown to play a critical role in increased stress and reduced cell survival (Müller-Rischart et al., 2013). Phenotypes caused by the knockdown of Parkin have been shown to mirror that of PINK1 deficiency in zebrafish, such as mitochondrial impairment and loss of dopaminergic neurons. Flinn et al. demonstrated for the first time that *Parkin* knockdown led to impaired mitochondrial complex I activity and reduced energy metabolism, as well as significantly reduced numbers of diencephalic dopaminergic neurons that were significantly more susceptible to the toxic effects of the mitochondrial neurotoxin MPTP. Cell loss was mostly restricted to the posterior tuberculum in zebrafish, an area anatomically homologous to the human substantia nigra (Flinn et al., 2009). However, a replicative study with *Parkin* knockdown to 50–60% neither recapitulated the loss of diencephalic dopaminergic neurons nor alterations in mitochondrial integrity, and the authors did not observe any morphological abnormalities or motor deficits in the morphant larvae (Fett et al., 2010). This discrepancy may result from different levels of remaining functional Parkin due to different knockdown methods. Nevertheless, Parkin-deficient larvae were found to be more sensitive to proteotoxic stress inducers, such as heat shock, leading to increased cellular apoptosis (Fett et al., 2010).

#### Park 7 and Park 15

Other genetic zebrafish models of PD include MO injections against the genes *Park 7* and *Park 15*, encoding DJ-1 and F-box only protein 7 (*FBXO7*). Patients with loss-of-function of the human *PARK7* gene that encodes DJ-1 present with early onset autosomal recessive PD (Bonifati et al., 2003). Zebrafish DJ-1 shares more than 80% conservation with human DJ-1 and is expressed throughout all dopaminergic neurons in the brain (Bai et al., 2006). While the knockdown of DJ-1 by itself did not lead to loss of dopaminergic neurons, DJ-1 morphants showed increased expression of apoptosis regulator genes such as *p53* and *Bax*, and were more susceptible to oxidative stress and neurotoxicity (Bretaud et al., 2007). Knockdown of the *FBXO7* ortholog (encoding PARK15) led to developmental abnormalities and deformations, significant loss of dopaminergic neurons, and behavioral as well as locomotor dysfunctions (Zhao et al., 2012).

#### Other models of Parkinson’s disease

In addition to the various genetic models of PD, several methods have been adopted to chemically induce neurotoxicity and the pathological hallmarks of PD in zebrafish (Table 2). One of the most common models is the administration of the toxin MPTP. In humans, MPTP exposure was found to induce dopaminergic neuronal cell loss and motor symptoms reminiscent of PD (Smeyne and Jackson-Lewis, 2005). Larval zebrafish were also found to be susceptible to MPTP treatment, resulting in the specific reduction of diencephalic dopaminergic neurons and significantly reduced locomotor activity (Bretaud et al., 2004). Several follow-up studies have since supported the use of MPTP to induce dopaminergic neuronal cell loss (Lam et al., 2005; McKinley et al., 2005; Wen et al., 2008; Sallinen et al., 2009) as well as locomotor deficits (Lam et al., 2005; Sallinen et al., 2009; Babu et al., 2016) in zebrafish. Using an enhancer trap transgenic fish line for the vesicular monoamine transporter 2 (*Vmat2*) gene, Wen et al. (2008) performed the first *in vivo* study to demonstrate selective loss of dopaminergic neurons following MPTP exposure. Subsequent studies in the field have adopted the zebrafish MPTP model in studies aiming to develop PD therapeutics. Investigating the potential of a platinum nanoparticle using leaf extract of a plant (BmE-PtNP), Nellore et al. (2013) showed that BmE-PtNPs were capable of ameliorating oxidative stress, rescuing both dopaminergic loss and motor deficits in MPTP-treated zebrafish larvae. Similarly, cyclopentylamino carboxymethylthiazolylindole (NecroX) compounds like the necrosis inhibitor-5 (NecroX-5) (Liu et al., 2015), as well as the iridoid glycoside loganin (Yao et al., 2017) exhibited neuroprotective potentials.

Furthermore, 6-OHDA administration in zebrafish resulted in selective loss of TH-positive dopaminergic neurons within days of application (Zhang et al., 2015, 2016; Cronin and Grealy, 2017), as well as locomotor impairments including reduced swim time and distance, and anxiety-related behavior (Feng et al., 2014; Zhang et al., 2015; Benvenutti et al., 2018). Direct intraventricular injection of 6-OHDA into the ventral diencephalon of adult fish also resulted in significant ablation of diencephalic dopaminergic neurons and locomotor deficits reminiscent of PD-related bradykinesia (Vijayanathan et al., 2017). However, significant recovery of neurons was observed within 30 days post-lesion.

Finally, to investigate the link between pesticides and PD, Lulla et al. (2016) used the pesticide ziram and demonstrated a γ1-synuclein-dependent mechanism leading to dopaminergic cell loss in zebrafish. While overexpression of γ1-synuclein resulted in intracytoplasmic neuronal aggregates and neurotoxicity, γ1-synuclein knockdown rescued the dopaminergic neuronal loss and motor impairments induced by ziram treatment (Lulla et al., 2016). Rotenone treatment of zebrafish embryos resulted in developmental retardation and deformities (Melo et al., 2015; Byrnes et al., 2018) accompanied by increased oxidative stress, cellular apoptosis and loss of dopaminergic neurons (Khotimah et al., 2015; Martel et al., 2015). In line with that, rotenone treatment induced locomotor deficits including markedly decreased motility and swim speed (Khotimah et al., 2015; Wang et al., 2017). Furthermore, using the light/dark box test approach, Wang et al. (2017) observed that zebrafish treated with rotenone spent more time in the light and showed longer latencies to enter the dark area, mirroring signs of anxiety and depression that are present in some PD patients. Even low doses of the pesticide paraquat impaired neurogenesis of dopaminergic neurons and caused motor and behavioral impairments in zebrafish embryos (Nellore and Nandita, 2015) along with increased expression of oxidative stress and apoptosis pathway components (Wang et al., 2016, 2018). Using a transgenic reporter line for macrophages, Wang et al. observed significant macrophage recruitment and migration following treatment with paraquat, providing evidence of immune cell reactivity in the paraquat-induced model of PD (Wang et al., 2016). Interestingly, experiments carried out by different groups on adult zebrafish using repeated intraperitoneal applications of paraquat resulted in conflicting results regarding motor deficits, anxiety and aggression (Bortolotto et al., 2014; Nunes et al., 2017).

## Alzheimer’s disease

Alzheimer’s disease (AD) is a chronic neurodegenerative disorder and the most frequent cause of dementia. AD is typically characterized by the presence of extracellular β-amyloid (Aβ) deposits generated from cleaved amyloid precursor protein (APP), as well as intracellular neurofibrillary tangles (NFTs) made up of aggregated hyperphosphorylated tau proteins. The disease leads to gradual hippocampal and parietal brain atrophy (Frisoni et al., 2010). GWAS (genome-wide association studies) have identified several high-risk loci genes that are involved in the regulation of immune responses (Lambert et al., 2013; dos Santos et al., 2017; McQuade and Blurton-Jones, 2019) suggesting a potential role for microglia in AD pathogenesis. Interestingly, Aβ plaques can be found in the brain even before the onset of cognitive decline. On the other hand, NFTs have been associated with neurodegeneration, cell death, and cognitive dysfunction (Bondi et al., 2017; Cass, 2017; DeTure and Dickson, 2019). As evidenced from recent PET imaging studies and meta-analyses of published biomarker data, a strong association between total tau levels in both cerebrospinal fluid and blood with cognitive impairment in AD patients has been detected (Hampel et al., 2018; Wattmo et al., 2020).

### Zebrafish models of Alzheimer’s disease

#### Tau

The first zebrafish tau model transiently expressing human tau protein in neurons showed tau hyperphosphorylation and accumulation in neuronal cell bodies and produced a cytoskeletal disruption that closely resembled the NFTs seen in human disease (Tomasiewicz et al., 2002; Table 3). Subsequently, stable zebrafish tau transgenic lines for the expression of human tau (111) or human mutant tau (Paquet et al., 2009) within the CNS were generated, which achieved significantly higher levels of tau expression and resulted in NFT-like tau accumulations. The success of these early tau transgenic models was validated by *in vivo* imaging that demonstrated rapid hyperphosphorylation and aggregation of tau as well as neuronal cell death (Paquet et al., 2009).

**TABLE 3:** Zebrafish models of tauopathy.

Study	Method(s)	Target of tau phosphorylation	NFT formation	Other phenotype
Bai et al., 2007	MAP-Tau4R mutation	Enolase-2 promoter – Neurons	Yes	
Cosacak et al., 2017	Tau P301L mutation	her4.1 promoter – NPCs (with radial glial identity) and neurons	No – Investigated in adult zebrafish	
Lopez et al., 2017	Tau A152T mutation	PanN:Gal4VP15 driver – Pan-neuronal	Yes	Increased neuronal cell death. Impaired proteasome function.
Paquet et al., 2009	Tau P301L mutation	HuC promoter – Neurons	Yes	Increased neuronal cell death. Reduced length and branching of motor neurons.
Tomasiewicz et al., 2002	FTDP-17 mutation	GATA-2 promoter – Neurons	Yes	Disruption of cytoskeletal filaments in cell axon

In a recent study, Lopez et al. (2017) generated a zebrafish line that expressed mutant A152T human tau pan-neuronally leading to increased cellular apoptosis, neurodegeneration, and impaired locomotor behavior in response to stimuli. Interestingly, despite effectively recapitulating the classical hallmarks of AD pathology, tau hyperphosphorylation and NFT formation were only observed in the spinal cord and not in the brain of the larval zebrafish (Tomasiewicz et al., 2002; Paquet et al., 2009; Fett et al., 2010; Lopez et al., 2017). Expression and hyperphosphorylation of mutant human P301L-tau in neuronal cells of adult fish did not result in neurodegeneration, nor formation of NFTs in the brain (Cosacak et al., 2017).

#### β-amyloid

The ‘amyloid hypothesis’ of AD was first proposed in the 1980s and posits that the neurotoxic build-up and deposition of Aβ aggregates is a major factor in AD pathogenesis (Glenner and Wong, 1984; Hardy and Allsop, 1991; Selkoe, 1991). However, it has since been demonstrated that Aβ accumulation in the brain is independent of the age at onset and severity of AD (Edison et al., 2007; Li et al., 2008; Fagan et al., 2009). Nonetheless, Aβ has a crucial role in AD pathology. In the zebrafish, Aβ plays a physiological role in the maintenance of healthy cerebrovascular functioning as increased levels of Aβ have been associated with aberrant cerebrovascular branching in the developing hindbrain (Cameron et al., 2012; Luna et al., 2013). Likewise, MO-mediated knockdown of APP levels and blocking of Aβ production in zebrafish larvae led to significant cerebrovascular defects (Luna et al., 2013). Since then, cerebroventricular microinjections of synthetic Aβ peptides have been adopted as the most direct approach to model AD pathology in the zebrafish to mirror Aβ aggregation, tau protein phosphorylation, and induced toxicity (Nery et al., 2014; Bhattarai et al., 2016, 2017; Table 4). Direct hindbrain ventricular injections of human Aβ finally recapitulated all the hallmarks of AD such as rapid formation of β-sheet aggregations, increased neuronal toxicity and cell death, as well as adverse motor functioning (Nery et al., 2014; Bhattarai et al., 2016). Interestingly, lithium chloride treatment reduced tau phosphorylation and ameliorated the motor deficits in the injected zebrafish (Nery et al., 2014).

**TABLE 4:** Zebrafish models of Aβ toxicity.

Study	Method(s)	Aβ aggregation	Neuronal cell death	NSPC proliferation and neurogenesis	Other phenotype
Bhattarai et al., 2016	Human Aβ 42 (ventricular injections)	Yes	Yes	Yes	Formation of intracellular Aβ-sheets. Impaired conditioning and reduced learned anxiety response
Bhattarai et al., 2017	Human TR-Aβ 42 (ventricular injections)	Yes	Yes	Yes	Increased microglia activation. Increased synaptic degeneration.
Nery et al., 2014	Aβ 1–42 (ventricular injections)	Not reported	Yes – But also observed in vehicle injected groups (compared to non-injected controls)	Not reported	Increased tau phosphorylation. Impaired avoidance of aversive stimulus.
Newman et al., 2010	Human Aβ 42 (expression in melanophores under mitfa promoter)	Not reported	Not reported	Not reported	Abnormal pattern and loss of skin pigmentation

However, zebrafish Aβ models also exhibited some phenotypes atypical of clinical AD progression. For example, Aβ overexpression increased neuronal progenitor plasticity and proliferation, and enhanced neurogenesis in the adult zebrafish brain *via* interleukin (IL)-4 secretion by neurons and activated microglia (Bhattarai et al., 2016, 2017). Interestingly, progenitor proliferation was preserved but neurogenesis diminished with aging (Nery et al., 2014). In a recent follow-up study by the same group, single-cell transcriptomic data demonstrated that IL-4 regulates serotonin production and downstream regulation of brain-derived neurotrophic factor (BDNF) expression leading to increased plasticity and proliferation of neural stem cells in the adult zebrafish brain (Bhattarai et al., 2020).

#### Presenilins

Another major focus in zebrafish AD research involves the *Presenilin* (*PSEN*) genes that have been implicated in the hereditary forms of AD (Strooper, 2007; Veugelen et al., 2016; Wong et al., 2020). PSEN is part of the γ-secretase complex involved in regulating cellular proliferation, and *PSEN* mutations have been suggested to play a role in the generation of plaque-building Aβ peptides (Haapasalo and Kovacs, 2011; Veugelen et al., 2016). During disease pathology, AD-related *PSEN* mutations have been shown to accelerate the catalytic proteolysis of APP by γ-secretase, resulting in the increased production of longer, amyloidogenic Aβ peptides (Kabir et al., 2020). Zebrafish carry two orthologs of the two different *PSEN* genes (*PSEN1* and *PSEN2*) found in the human genome and these are ubiquitously expressed throughout development (Leimer et al., 1999; Groth et al., 2002). Early *in vitro* work found that human *PSEN1* could be replaced by zebrafish *Psen1*, maintaining the efficacy of generating Aβ from APP (Leimer et al., 1999). While complete *Psen1* KO (*Psen1*^–/–^) was found to be lethal in mice, *psen1*^–/–^ zebrafish were viable and did not exhibit gross morphological defects when compared to wild type zebrafish (Sundvik et al., 2013). Intriguingly, MO-mediated knockdown of *Psen2* led to significant apoptosis and neuronal loss in zebrafish (Campbell et al., 2006), while *Psen2*^–/–^ mice only exhibited a weak phenotype (Herreman et al., 1999). Impaired Notch signaling could be recapitulated following the loss-of-function of either of both PSEN genes in the zebrafish (Nornes et al., 2003, 2008, 2009; Campbell et al., 2006; Sundvik et al., 2013). Notch signaling is often affected in AD pathology, and regulates the histamine system in the brain and the development of histaminergic neurons (Zlomuzica et al., 2016).

#### Other models of Alzheimer’s disease

One of the most widely used methods to pharmacologically induce an AD-like phenotype is through okadaic acid (OKA) application. OKA is a polyether C38 fatty acid toxin and a potent and selective inhibitor of the protein phosphatases 1 (PP1) and 2A (PP2A) (Kamat et al., 2014; Kamat and Nath, 2015). OKA-induced inhibition of these protein phosphatases, in particular PP2A, has previously been shown to cause tau hyperphosphorylation and NFT formation, as well as other hallmarks of AD pathology both *in vitro* and *in vivo* (Hensley et al., 2010a; Zhang and Simpkins, 2010; Kamat et al., 2012a,b, 2013, 2014; Kamat and Nath, 2015). Exposure of zebrafish to OKA resulted in tau hyperphosphorylation, as well as deposition of Aβ and formation of senile plaques (Nada et al., 2016). Furthermore, exposure to OKA induced learning and memory deficits in zebrafish as revealed by the pre-treatment learning and post-treatment memory test developed by Williams et al. (2002), Administration of lanthionine ketimine-5-ethyl ester, shown to elicit neurotrophic and neuroprotective properties in murine models (Hensley et al., 2010a,b) led to reduced cell apoptosis, increased BDNF production and rescue of the learning deficits of OKA-treated fish (Koehler et al., 2018). The efficacies of glycogen synthase kinase 3β (GSK3β) inhibitors have also been tested in zebrafish AD models. GSK3β is a serine/threonine protein kinase that has been involved in AD pathogenesis. Its overexpression in zebrafish is associated with increased Aβ production, tau hyperphosphorylation, neuronal cell death, reactive gliosis, and cognitive impairments (Hooper et al., 2008; Llorens-Marítin et al., 2014; Jaworski et al., 2019; Toral-Rios et al., 2020). Supporting an earlier *in vivo* study, a recent investigation of the selective GSK3β inhibitor, TDZD-8, in zebrafish revealed that the reduced GSK3β activity was associated with reduced tau phosphorylation and mortality rates. Importantly, treatment with TDZD-8 also rescued OKA-induced cognitive deficits (Paquet et al., 2009; Koehler et al., 2019).

## Amyotrophic lateral sclerosis (Lou Gehrig’s disease)

Amyotrophic lateral sclerosis also known as motor neuron disease (MND) or Lou Gehrig’s disease, is a progressive neurodegenerative disease of the human motor system (Kiernan et al., 2011; Brown and Al-Chalabi, 2017). ALS is relatively rare with incidence rates of only 2–3 per 100,000 individuals worldwide, and remains an incurable, fatal disorder. Characterized by loss of primary motor neurons in the brain and spinal cord, ALS leads to progressive spasticity and muscle atrophy, dysarthria and dysphagia, and ultimately paralysis and death (Loeffler et al., 2016; Grad et al., 2017; Matsubara et al., 2019). ALS is categorized into two forms, sporadic ALS that makes up approximately 90% of all cases, and familial ALS (Kiernan et al., 2011; Brown and Al-Chalabi, 2017). More than 30 different genes have been associated with familial ALS (Hardiman et al., 2017). Of these, mutations in four genes are the most prevalent and contribute to up to 70% of all familial ALS cases (Hardiman et al., 2017). They comprise the mutations in *SOD1* (superoxide dismutase 1), *TARDBP* (encoding TAR DNA-binding protein-43 [TDP-43]), *C9orf72* (open reading frame 72 on chromosome 9), and *FUS* (Fused in Sarcoma) (Al-Chalabi et al., 2017; Roggenbuck et al., 2017).

### Zebrafish models of amyotrophic lateral sclerosis

The transparent bodies of zebrafish larvae allow for non-invasive visualization of motor neurons, from somas to their neuromuscular synapses, as well as their circuitry. This and the availability of genome editing, optogenetics and ease of drug screening facilitate functional analyses of ALS-associated proteins and consequential motor neuron degeneration with subcellular resolution (Asakawa et al., 2021).

The genes most commonly associated with ALS show high conservation in zebrafish with sequence identity of over 70% for *SOD1* (Da Costa et al., 2014), *TARDBP* (Kabashi et al., 2011), *C9orf72* (Iyer et al., 2018), and *FUS* (Morrice et al., 2020). Stable mutant zebrafish lines have been established for all four of these genes (Tables 5–8), and all effectively recapitulate the various hallmarks observed in ALS.

**TABLE 5:** Zebrafish models of SOD1 pathology.

Study	Method(s)	Axonopathy	MN loss	NMJ abnormalities	Motor deficits	Other phenotypes
Benedetti et al., 2016	SOD1 G93R mutation	Yes – Reduced primary and unbranched axonal length, and increased aberrant branching	Yes	No	Yes – Reduced swim distance and duration	Reduced innervation and muscle atrophy. Increased inflammation and reactive astrogliosis.
Da Costa et al., 2014	TILLING-mediated SOD1 T70I missense mutation	Not reported	Yes	Yes – Reduced colocalization of SV2 and α-bungarotoxin in the interseptal region	Yes – Reduced swim duration and velocity	
Lemmens et al., 2007	SOD1 G93A, G37R, and A4V mutations	G93A: Yes – 64.6% of injected. G37R: Yes – 68.8% of injected. A4V: Yes – 73.1% of injected.	Not reported	Not reported	Not reported	
Ramesh et al., 2010	BAC-mediated SOD1 G93R mutation	No	Yes	Yes – Reduced colocalization of SV2 and α-bungarotoxin, as well as NMJ volume (in adults)	Yes – Reduced swim endurance (in adults)	Muscle atrophy. Progressive and intermittent paralysis. Increased mortality.
Sakowski et al., 2012	SOD1 G93A mutation	Yes – Reduced axon length and increased branching	Yes	Yes – Loss of intact NMJ (from 20 weeks)	Yes – Reduced swim velocity and progressive reduced swim duration	

**TABLE 6:** Zebrafish models of TARDBP (TDP-43) pathology.

Study	Method	Axonopathy	MN loss	NMJ abnormalities	Motor deficits	Other phenotypes
Bose et al., 2019	CRISPR/Cas9-mediated knockout	Not reported	Not reported	Yes – Abnormal structure and deficits in pre- and postsynaptic NMJ transmission	Yes – Reduced swim duration, distance, and maximum velocity	Morphological deformities. Increased mortality.
Hewamadduma et al., 2013	TILLING-mediated TARDBP fh301 (Y220) missense mutation	No (not in *tardbp*^fh301/fh301^ mutants) – But severe axonal defects in double (*tardbp* + *tardbpl*) knockouts	Not reported	Not reported	Yes – In double (*tardbp* + *tardbpl*) knockout	Morphological abnormalities and increased mortality in double (*tardbp* + *tardbpl*) knockouts
Kabashi et al., 2010	AMO knockdown	Yes – Reduced axon length and aberrant branching	No	Not reported	Yes – Impaired tail coiling ability and loss of TEER	
Kabashi et al., 2010	TARDBP A315T, G348C, and A382T mutations	A315T: Yes – Reduced axon length and increased branching. G348C: Yes – Reduced axon length and increased branching. A382T: Yes – Reduced axon length	A315T: Yes – 48%. G348C: Yes – 44%. A382T: Yes – 31%.	Not reported	Yes – Impaired tail coiling ability and delayed response to TEER	
Laird et al., 2010	TARDBP A315T mutation	Yes – Reduced axon length and increased aberrant branching	Not reported	Not reported	Not reported	
Lissouba et al., 2018	TARDBP G348C mutation	Yes – Increased aberrant branching of primary axon and absence of secondary branching	Not reported	Not reported	Yes – Deficient TEER; reduced swim distance, duration, and maximum velocity	
Schmid et al., 2013	TARDBP double (*tardbp*^–/–^*; tardbpl*^–/–^) mutation	Yes – Reduced axon length	Not reported	Not reported	Not reported	Impaired blood circulation. Muscle atrophy. Increased mortality.

**TABLE 7:** Zebrafish models of FUS pathology.

Study	Method	Axonopathy	MN loss	NMJ abnormalities	Motor deficits
Armstrong and Drapeau, 2013	Antisense MO knockdown (60% expression reduction)	Not reported	Not reported – But increased MN excitability	Yes – Abnormal structure and deficits in pre- and postsynaptic NMJ transmission	Yes – Reduced swim duration and increased fatigue
Armstrong and Drapeau, 2013	FUS R521H mutation	Not reported	Not reported – But increased MN excitability	Yes – Abnormal structure and deficits in pre- and postsynaptic NMJ transmission	Yes – Reduced swim duration and increased fatigue
Armstrong et al., 2016	CRISPR/Cas9-mediated knockout	Not reported	Not reported	Not reported	Not reported
Kabashi et al., 2011	AMO knockdown	Yes – Reduced primary and unbranched axonal length	Not reported	Not reported	Yes – Deficient TEER (57% of injected)
Kabashi et al., 2011	FUS R521C, R521H, and S57Δ mutations	R521H: Yes – Reduced axonal length. R521C: No S57Δ: No	Not reported	Not reported	R521H: Yes – Deficient TEER (57% of injected). R521C: No S57Δ: No
Lebedeva et al., 2016	CRISPR/Cas9-mediated knockout	No	Not reported	Not reported	No

**TABLE 8:** Zebrafish models of C9orf72 pathology.

Study	Method	Axonopathy	MN loss	Motor deficits	Other phenotypes
Ciura et al., 2013	AMO knockdown	Yes – Reduced motor neuron axon length and aberrant branching	Not reported	Yes – Deficient touch-evoked escape response (TEER); reduced swim distance, average velocity and maximum velocity	
Lee et al., 2013	8×, 38×, and 72× GGGGCC repeats	Not reported	Not reported	Not reported	Increased apoptotic cell death in 38× and 72× embryos
Ohki et al., 2017	2× or 80× GGGGCC repeats (with or with ATG codon)	No	Not reported	Not reported	Display of toxicity and pericardial edema in 80× embryos
Shaw et al., 2018	89× GGGGCC repeats	Not reported	Yes	Yes – Impaired ability to transition into fast movement. Displayed center avoidance behavior.	Muscle atrophy. Reduced weight gain. Increased mortality.
Swinnen et al., 2018	3×, 4×, 10×, ∼35×, ∼70×, and ∼90× GGGGCC repeats	Yes – Only from ∼35× repeats	Not reported	Not reported	

#### Superoxide dismutase 1

Mutations in *SOD1* are a major cause of familial ALS, and one of the most extensively studied. Interestingly, *SOD1* expression is ubiquitous and despite efforts to ascertain its role, it is still unclear how mutant SOD1 leads to selective death of motor neurons. Evidence presented thus far suggests that pathology arises from gain-of-function mutations of SOD1 in ALS. As summarized in Table 5, overexpression of human mutant *SOD1* in zebrafish resulted in significantly impaired axonal outgrowth and limited branching, as well as impaired swimming capabilities. In one of the first studies, transient overexpression of human *G93A-SOD1* caused axon pathology, degeneration of the neuromuscular junctions, and motor neuron loss (Sakowski et al., 2012). Similarly, zebrafish larvae injected with mutant human *SOD1* mRNA showed significant axonopathy and displayed impaired movements during behavioral tests when compared to controls injected with wild type *SOD1* mRNA (Robinson et al., 2019). These phenotypes were confirmed in various stable transgenic zebrafish models of mutant SOD1 (Ramesh et al., 2010; Sakowski et al., 2012; Benedetti et al., 2016).

#### TARDBP

*TARDBP* encodes the RNA/DNA-binding protein TDP-43 which functions in RNA processing and metabolism. *TARDBP* mutations result in accumulation of mutant TDP-43 in inclusion bodies in over 90% of all ALS cases. Also present in frontotemporal lobe dementia (FTLD), ubiquitinated and hyperphosphorylated TDP-43 C-terminal fragments accumulate in neurons and glia in brains of FLTD patients, but interestingly not in the spinal cord (Berning and Walker, 2019).

Zebrafish transgenic for human mutant *TARDBP* exhibit hallmarks of ALS pathogenesis such as abnormalities in motor axon formation and branching, and motor functioning (Table 6). Testing the effects of three separate *TARDBP* mutations (A315T, A382T, and G348C) found in ALS patients, Kabashi et al. (2010) showed for the first time that mutant TDP-43 expression in the zebrafish led to abnormal motor axon development, motor neuron defects and toxicity, as well as severe deficits of motor function. In a follow-up study, Laird et al. (2010) demonstrated that overexpression of *proganulin*, null mutations of which cause TDP-43 accumulation in FTLD, rescued motor neuron degeneration in the TDP43-A315T zebrafish line. Zebrafish express both independent orthologs of *TARDBP* (*tardbp* and *tardbpl*) throughout development, and single deletion of one ortholog is compensated for by increased expression of a splice variant of the other (Schmid et al., 2013). Therefore, homozygous double mutants (*tardbp*^–/–^*; tardbpl*^–/–^) were generated to establish the impact of TDP-43 loss-of-function (Schmid et al., 2013; Bose et al., 2019). *tardbp*^–/–^*; tardbpl*^–/–^ zebrafish exhibited mispatterned vasculature, impaired outgrowth of spinal motor neuron axons, and smaller muscle fibers with disorganized assembly of myofibrils (Schmid et al., 2013). Expression of Filamin C was also upregulated in the *tardbp*^–/–^*; tardbpl*^–/–^ double mutants. Filamin C is an actin cross-linking protein found in smooth muscle cells surrounding the brain vasculature, and analysis of Filamin C expression in frontal cortex of FTLD-TDP patients confirmed its upregulation as part of TDP-43 mediated pathology (Schmid et al., 2013). Further analyses also found aberrant quantal transmission and perturbed synapse architecture at the NMJ in the double mutants and highlighted different sensitivity to therapeutic drugs in partial vs. complete loss of allelic functions of TDP-43 (Bose et al., 2019).

#### Fused in sarcoma

Mutations in the *FUS* gene cause an aggressive, sometimes juvenile-onset motor neuron disease (Lattante et al., 2013). FUS is an RNA-binding protein with structural and functional similarity to TDP-43, and mis-localization of FUS from the nucleus into the cytoplasm leads to FUS aggregation in inclusion bodies similar to TDP-43 inclusions in motor neurons. As with the other ALS-associated genes, *FUS* has been suggested to contribute to disease pathogenesis through either a loss-of-function in the nucleus or gain-of-function after its mis-localization to the cytoplasm. As summarized in Table 7, several zebrafish models with genetic manipulation of *FUS* have been developed. The earlier studies employed antisense MOs to knockdown *FUS*, resulting in morphant larvae with shortened motor axons, aberrant branching and marked abnormality in motor behavior (Kabashi et al., 2011; Armstrong and Drapeau, 2013). In the same studies, the authors also investigated the possible toxic gain-of-function of FUS by overexpression of the human gene carrying the ALS-causing R521H mutation. This resulted in significant axonopathy, increased motor neuron excitability, NMJ abnormalities, as well as impaired motor function (Kabashi et al., 2011; Armstrong and Drapeau, 2013). To investigate the mechanism by which mutations in *FUS* cause motor neuron degeneration, Yu et al. examined the role of U1 snRNP (Yu et al., 2015), a small nuclear ribonucleoprotein that had been shown to be the most abundant factor interacting with FUS in previous *in vitro* assays (Yamazaki et al., 2012; Groen et al., 2013). They found that FUS is essential for the interaction between U1 snRNP and RNA Polymerase II (RNAP II) during splicing, thus physically and functionally coupling transcription to splicing (Yu et al., 2015). MO-mediated knockdown of U1 snRNP in zebrafish led to the development of significantly truncated motor axons in the developing zebrafish larvae (Yu et al., 2015). Confirming that the pathological impact of *FUS* mutations seems to lie in its impaired function or mislocalized gain of function, full deletion of zebrafish *FUS* employing CRISPR/Cas9 resulted neither in visible defects of axonal length or branching, nor any abnormality of swimming behavior (Lebedeva et al., 2016). The absence of a phenotype typical for ALS upon *FUS* deletion in zebrafish is in line with previous findings in *FUS* knockout mouse models that displayed a range of phenotypes atypical of ALS (Hicks et al., 2000; Kino et al., 2015).

#### C9orf72

Expansions of the G4C2 (GGGGCC) hexanucleotide repeats in the *C9orf72* gene have been identified as one of the most common genetic causes of ALS (Stepto et al., 2014; Balendra and Isaacs, 2018). While healthy individuals commonly have up to 30 G4C2 repeats, ALS patients carry versions of 70 or more G4C2 repeat expansions. While expansion mutations of *C9orf72* are mostly responsible for the detrimental effects on motor neuron degeneration, loss of function resulting from *C9orf72* haploinsufficiency has also been suggested to induce neurodegeneration.

Various mutant, deletion and transgenic C9orf72 zebrafish models have since been generated to investigate the functional role of C9orf72 in ALS pathogenesis (Table 8). Multi-fold G4C2 repeats were engineered to achieve neurotoxicity and induce disease. In one of the early studies, increased apoptotic cell death was observed in embryos expressing 72× G4C2 repeats compared to age-matched embryos with 38× G4C2 repeats (Lee et al., 2013). In line with data suggesting correlating increase in neurotoxicity with numbers of G4C2 repeats, it was found that injection of 89× G4C2 repeats at the 1-cell -stage led to severe toxicity and mortality by 7 dpf (Shaw et al., 2018). Expression of 10 or fewer G4C2 repeats did not have any negative impact on axon physiology (Swinnen et al., 2018). The zebrafish models with multiple G4C2 expansions displayed hallmarks of ALS such as muscle atrophy, motor axon abnormalities, motor neuron loss, as well as behavioral and locomotor deficits.

The first *in vivo C9orf72* loss-of-function model was developed *via* a MO-induced knockdown leading to signs of motor neuron degeneration with axonopathy phenotypes and abnormal motor behavior (Ciura et al., 2013). Recent studies mimicking C9orf72 loss of function involve the generation of novel transgenic lines which include the specific deletion of the functional DENN domain of C9orf72 (Yeh et al., 2018) as well as a miRNA-based gene-silencing approach to achieve loss of over 50% C9orf72 function (C9-miR) (Butti et al., 2020). The reduced C9orf72 expression resulted in aberrant axonal growth, increased neuronal apoptosis and muscle atrophy, as well as and locomotor deficits when compared to age-matched controls.

Furthermore, mislocalization of TDP-43 from the nucleus to the cytoplasm was observed in the C9-miR zebrafish, recapitulating another classical neuropathological hallmark of ALS (Butti et al., 2020).

#### Other models of amyotrophic lateral sclerosis

Besides genetic causes, extrinsic factors such as the exposure to environmental neurotoxins have been associated with ALS etiology (Zarei et al., 2015). Bisphenol A (BPA) is widely used in the production of polycarbonate plastics and adversely affects hormonal and metabolic pathways (Vandenberg et al., 2007; Seachrist et al., 2016). Morrice et al. (2018) tested the effects of synthetic estrogen BPA on zebrafish and found that BPA led to reduced motor axon length and branching as well as loss of neuromuscular junction integrity. They further noted increased numbers of apoptotic motor neurons and the presence of activated microglia.

## Huntington’s disease

Huntington’s disease (HD) is an autosomal-dominant inheritable neurodegenerative disorder that results from a CAG repeat extension in exon 1 of the huntingtin gene (HTT), which translates into a long polyQ repeat in the huntingtin protein. The length of these polyQ repeats correlate with the age of onset, penetrance and severity of the disease (Langbehn et al., 2010). HD has a prevalence of around 10 per 100,000 individuals globally, with the age of onset, penetrance, and severity of the disease largely dependent on the number of CAG/polyQ repeats present in the huntingtin gene. Mutant HTT (mHTT) fragments are very unstable as a consequence of the long polyQ repeat and forms aggregates that are localized in intranuclear inclusions, a hallmark of HD pathology (Squitieri et al., 1994; Lee et al., 2012; Semaka et al., 2013). Impaired proteostasis of mHTT result in toxic effects leading to neuronal death of GABAergic medium spiny neurons in the striatum and in neuronal loss in the cortex and other brain regions (Eidelberg and Surmeier, 2011; Ehrlich, 2012; Estrada-Sánchez and Rebec, 2013; Garret et al., 2018; Hsu et al., 2018). Despite having been first described by George Huntington over a century ago, the disease remains incurable. No treatment options are currently available to prevent or slow down the progression of HD, with the average patient succumbing to the disease within 20 years from onset of disease symptoms including behavioral, cognitive, as well as motor dysfunctions (Bates et al., 2015; McColgan and Tabrizi, 2018).

### Zebrafish models of Huntington’s disease

#### Htt loss-of-function

To identify the physiological function of Htt, various groups have addressed the impact of *htt* depletion on early development in the zebrafish (Table 9). The zebrafish homolog of human *HTT* encodes a protein of 3,121 amino acids with 70% identity to mammalian HTT, but only 4 glutamines compared to 7 in mice and up to 35 in humans (Karlovich et al., 1998). As in humans, HTT is ubiquitously expressed in the zebrafish brain and is crucial for the formation of telencephalic progenitor cells as well as pre-placodal cells (Lumsden et al., 2007; Henshall et al., 2009). The zebrafish telencephalon has been proposed to be the anatomical equivalence of the mammalian striatum (Rink and Wullimann, 2002). Moreover, the loss of placode-derived tissue including olfactory and lateral line sensory neurons in the zebrafish is consistent with clinical observations of progressive olfactory abnormalities in individuals with HD (Mitchell et al., 2005; Laroche et al., 2020).

**TABLE 9:** Zebrafish models of huntingtin pathology.

Study	Method	Neuronal loss	Impaired metabolism	Motor deficits	Other phenotype
Diekmann et al., 2009	AMO knockdown	Yes	Reduced BDNF levels.	Not reported	Morphological deformities. Increased mortality.
Henshall et al., 2009	AMO knockdown	Too early	Not reported	Not reported	Impaired brain development. Morphological deformities.
Lo Sardo et al., 2012	AMO knockdown	Not reported	Increased ADAM10 activity. Increased Ncadherin cleavage.	Not reported	Impaired brain development.
Lumsden et al., 2007	AMO knockdown	Not reported	Impaired iron metabolism. Reduced hemoglobin production.	Not reported	Developmental retardation and morphological deformities.
Miller et al., 2005	19Q, 35Q, 56Q, and 80Q polyQ expansion	Yes – Only from 56Q expansion	Not reported – But demonstrated role of CHIP in role of QC	Not reported	Morphological deformities and increased mortality (From 56Q expansion).
Schiffer et al., 2007	4Q, 25Q, and 102Q polyQ expansion	Yes – Only in 102Q	Not reported	Not reported	Morphological deformities and increased mortality (102Q).
Sidik et al., 2020	CRISPR/Cas9 deletion	No	No	Not reported	Reduced fitness and survival in adulthood.
Veldman et al., 2015	Cre-loxP inducible 97Q expansion – in relation to N17 domain	Not reported – But increased mHTT aggregation	Not reported	Yes	Brain atrophy and reduced brain weight (mHTT without N17 domain).

Focusing on regions of HTT expression throughout the CNS, Henshall et al. investigated how loss of HTT affected different brain regions in the zebrafish (Henshall et al., 2009). Inhibition of HTT mRNA translation impaired the formation of the anterior-most region of the neural plate, evidenced by reduced expression of genes characteristically expressed within that region (six1, dlx3b, and emx3). The anterior neural plate is important for the induction of forebrain structures such as the pre-placodal cells and telencephalic precursors (Whitlock and Westerfield, 2000; Andoniadou and Martinez-Barbera, 2013; Schmidt et al., 2013). In an independent follow-up study, Lo Sardo et al. investigated HTT function in neural tube formation. In concurrent experiments with htt-null mouse embryonic stem cells and HTT MO depleted zebrafish embryos, they found that HTT is essential for homotypic interactions between neuroepithelial cells (Lo Sardo et al., 2012). Inhibition of HTT translation resulted in impaired rosette formation and neurulation, similar to N-Cadherin ablation (Lele et al., 2002). HTT was also found to be required for proper expression and distribution of the apical marker ZO-1, the impairment of which resulted in mispositioned cells in the diencephalic neural tube and cellular aggregates in the brain ventricles at 24 h post fertilization (Lo Sardo et al., 2012). Furthermore, *hTT* morphants exhibited altered ventricular space and reduced cephalic regions compared with controls. Interestingly, as in N-Cadherin mutants (Lele et al., 2002), the impact of HTT depletion was restricted to alar regions in the forebrain, with cells remaining organized in more basal positions.

Knockdown of HTT also led to decreased hemoglobin in the blood, increased erythroid and ubiquitous transferrin receptor transcript levels, as well as exhausted maternal iron stores in the yolk (Lumsden et al., 2007). In line with iron deficiency and dysregulation of iron metabolism observed in human HD pathology (Morrison and Nevin, 1994), the results suggested that HTT acts downstream of transferrin receptor -mediated endocytosis of iron, thus implicating its role in the release of iron from endocytic compartments into the cytosol.

Brain-derived neurotrophic factor (BDNF) is a major contributor in neural development including formation, differentiation, and survival of neurons (Cohen-Cory et al., 2010; Kowiański et al., 2018). Wild type HTT increases the expression of *bdnf* at the transcriptional level. In the brain, HTT retains the transcription factor REST (repressor element 1 silencing transcription factor) in the cytoplasm, thereby reducing its repressor function of REST-regulated gene transcription and in turn facilitating BDNF expression (Gopalakrishnan, 2009). Post-mortem samples of HD brain revealed translocation of REST into the nucleus, leading to reduced transcription of BDNF (Zuccato et al., 2003, 2007). Similar to what has been seen in mouse models (Zuccato et al., 2001; Cattaneo et al., 2005) the effect of HTT knockdown in zebrafish causes reduced expression levels of BDNF to about 50% (Diekmann et al., 2009). This caused a high rate of neuronal apoptotic cell death and small eyes and head, enlargement of brain ventricles and later lower jaw abnormalities with missing branchial arches. All these observed phenotypes caused by knock down of zHTT could be rescued following treatment with recombinant BDNF administered through the embryo medium. Similarly, Henshall et al. found the defects in sensory nerve cell numbers of the lateral line descending from placodal cells they observed in HTT morphant embryos were rescued following treatment with exogenous BDNF (Henshall et al., 2009). By combining virtual screening for compounds that disrupt the REST transcription repressor complex and zebrafish *in vivo* experiments, Conforti et al. (2013) identified quinolone-like compound 91 (C91) as being able to interfere with the REST transcriptional machinery, thereby rescuing *BDNF* mRNA expression in zHTT morphants as well as embryos transiently expressing human N-terminal mHTT.

#### Htt gain-of-function

The accumulation of mHTT in neurons indicates that protein quality control is compromised. The C-terminus of Hsc70-interacting protein (CHIP) plays a crucial role in ensuring the proper folding and conformation of proteins, linking molecular chaperones with the ubiquitin-protease system by acting as both co-chaperone and ubiquitin ligase (McDonough and Patterson, 2003). The co-expression of CHIP with either a generic polyQ-fragment or pathogenic fragment of human HTT led to increased solubility and hence reduced aggregation of the mHTT protein in zebrafish (Miller et al., 2005). Furthermore, CHIP expression rescued the formation of inclusions in neurons and toxicity in mouse primary neuron cultures as well as zebrafish embryos (Miller et al., 2005). However, the apoptotic neuronal cells observed in mHTT expressing embryos were not the cells showing insoluble inclusion bodies of aggregated mHTT (Schiffer et al., 2007; Diekmann et al., 2009). In fact, soluble oligomers were highly interactive with RNA-binding proteins as well as proteins functioning in ribosome biogenesis, translation, transcription, and vesicle transport, whereas insoluble inclusions were less interactive and associated strongly with protein quality control components (Kim et al., 2016).

Exploiting the advantage of the zebrafish model for chemical screens, Schiffer et al. tested several compounds to inhibit polyQ aggregation of mutant HTT. Two anti-prion compounds of the N′-benzylidene-benzohydrazide class were identified that successfully inhibited polyQ aggregation in zebrafish embryos expressing mHTT with a 102Q (Schiffer et al., 2007). Williams et al. used the rhodopsin promoter to express an EGFP tagged mHTT containing a 71Q expansion in the zebrafish eye. This resulted in the accumulation of mHTT aggregates in the embryonic zebrafish retina, leading to loss of rhodopsin expression and consequential rod photoreceptor degeneration (Williams et al., 2008). In addition, the group performed an *in vitro* chemical screen to identify novel enhancers of autophagy-inducing pathways independent of mTOR (mammalian target of rapamycin). Autophagy is a major clearance mechanism for intracellular protein aggregates which can be upregulated *via* the administration of the mTOR inhibitor rapamycin (Ravikumar et al., 2004). Treatment of embryos expressing mHTT (71Q) with various drug candidates that all targeted different components of an mTOR-independent autophagy pathway (verapamil, calpastatin, clonidine, and 2′5′ddA), reduced the extend of mHTT aggregation in the retina significantly and substantially enhanced rhodopsin expression compared to untreated fish (Williams et al., 2008).

As previously reported in HD BAC transgenic mice, an N-terminal 17 (N17) amino acid fragment of HTT adjacent to the polyQ expansion domain regulates protein stability, toxicity, and sub-cellular localization (Gu et al., 2015). Investigating the role of N17 in zebrafish, Veldman et al. (2015) generated a novel, inducible line that expressed either a deleted N17 domain coupled with 97Q expansion (*mHTT-*Δ*N17-exon1*) or intact N17 and 97Q expansion alone (mHTT-exon1). The mHTT-ΔN17-exon1 embryos showed robust and rapidly progressive movement disorders, reminiscent of that seen in other animal models and human patients while the *mHTT-exon1* embryos displayed a delayed onset and slower progression of movement deficits. Importantly, accumulation of mutant HTT was observed in neurons expressing the combined N17 deletion and 97Q expansion, but not in neurons expressing the 97Q expansion construct alone (Veldman et al., 2015). Recently, a CRISPR/Cas9-induced *Htt* deletion was performed in zebrafish (Sidik et al., 2020), and in contrast to previous MO studies, and rodent models whereby homozygous HTT knockout is embryonic lethal at pre-gastrulation stage (Nasir et al., 1995; Zeitlin et al., 1995), these zebrafish were viable into adulthood but showed slow growth, smaller body size and poor fitness. Thus, further investigation is needed to establish the function of wild type Htt during development and the pathogenic role of the poly-Q expansion in zebrafish.

## Discussion

The zebrafish model is widely used to study various aspects of the CNS, from development and physiological function to degeneration and disease. We have highlighted many advantages, but we also want to address areas where zebrafish models show limitations.

Many of the studies summarized here, especially those dating 10 or more years back, have used MO to knock down mRNA or block protein expression. In general, the advantage of gene knockdown by MO injection has been the quick generation of larval fish depleted of proteins of interest but some of the produced phenotypes were caused by unspecific side effects. In recent years, many studies have dealt with the discrepancies between phenotypes of morphants and mutations leading to gene deletion or premature stop codons (Kok et al., 2015; Morcos et al., 2015; Stainier et al., 2015; Zimmer et al., 2019). MOs have been shown to induce off-target effects that were mostly morphological deformities, while engineered gene mutations generating nonsense-mRNAs were found to activate nonsense-mediated decay machinery and induce genetic compensation, masking the null mutant phenotype (Kok et al., 2015; El-Brolosy et al., 2019). Another obvious difficulty concerning the use of MOs to model human disease is their transient nature, as MOs only last up to 6 days of early development. The argument is often raised against the use of zebrafish that analysis is usually confined to the early transparent larval stages and not adult fish, even if the CNS is fully formed at 3dpf. However, adult fish can be employed for analysis, as demonstrated by Vijayanathan et al. (2017) who injected the neurotoxin 6-OHDA into the ventral diencephalon resulting in significant ablation of diencephalic dopaminergic neurons and locomotor deficits reminiscent of PD-related bradykinesia. Although zebrafish may intuitively not be the first choice to model late onset human disease and degenerative pathologies due to aging, we highlight in this article that many aspects of neurodegeneration can be successfully modeled and investigated in zebrafish larvae. Also, results from gene function analyses in early developmental stages may present clues about the predisposition for certain pathologies and the premanifest stages of disease. With the advent of CRISPR/Cas gene editing in zebrafish, the limitations of MO use have been widely overcome. In addition, the development and availability of pigmentation mutants, such as the *casper* (*nacre*^w2/w2^*; roy*^a9/a9^) and *crystal (nacre*^w2/w2^*; alb*^b4/b4^*; roy*^a9/a9^) mutants, may be of use to future investigations that require *in vivo* imaging in adult zebrafish (Antinucci and Hindges, 2016).

Motor dysfunction is a hallmark of many neurodegenerative disorders and is often used as a readout for disease progression. To observe and analyze motor function in zebrafish, imaging and tracking software have been developed to determine locomotor dysfunction. However, most studies have evaluated motor deficits through either total locomotor activity (swim duration, swim velocity, swim distance) or response time toward a tactile-evoked escape response. These tests may not be a true reflection of the more subtle muscular and movement disorders resulting from neurodegeneration. For example, it is difficult to establish balance or movement coordination in the zebrafish model, whereas it is straightforward in quadruped rodent models. Similarly, while anxiety and fatigue tests, as well as gait analyses have been routinely carried out in rodents, those assays have been more difficult to establish in the zebrafish model. Therefore, a need remains for the design of behavioral assays in zebrafish that more accurately mirror the repertoire of progressive motor disorders in neurodegenerative disorders.

**FIGURE 2:**
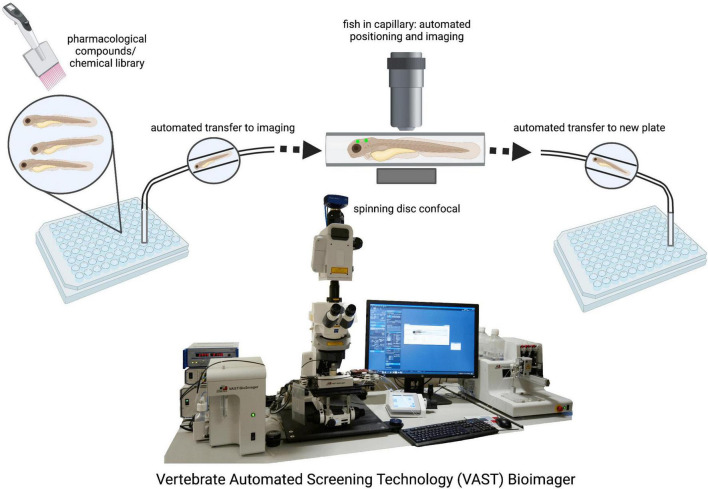
Drug screening and automated imaging as well as analysis of zebrafish larvae. The VAST system is a fully validated, highly accurate integrity screening system that provides a method of screening large population numbers rapidly. Automated imaging and analysis pipelines ensure unbiased and fast data generation. VAST Bioimager photo provided by Dr. Jason Early (Jason.Early@ed.ac.uk).

The ability to maintain large stock numbers of zebrafish at relatively low cost provides laboratories a cost-effective alternative to the breeding of rodents and other mammalian models (Aleström et al., 2019). The zebrafish can provide researchers with a platform for rapid high throughput readouts with large sample sizes which is particularly beneficial in studies involving clinical drug discovery as hundreds of samples can be placed into 96-well plates for simultaneous chemical screening (Letamendia et al., 2012; Mathias et al., 2012; Figure 2). Recent advances in microscopy facilitate high throughput bulk imaging in combination with compound screens in zebrafish larvae *in vivo*, such as the VAST (Vertebrate Automated Screening Technology) BioImager system developed for the zebrafish (Figure 2; Pulak, 2016; Guo et al., 2017; Early et al., 2018). However, while studies have demonstrated the efficacy of drug testing in the zebrafish to ameliorate phenotypes in the various models of neurodegenerative disorders, the method of drug administration is not entirely controllable. Treatment is usually carried out through the application of the respective drugs into the swimming media, and while experimental concentrations can be calculated, it remains difficult to accurately determine the amount that is absorbed by the zebrafish. Moreover, it remains to be established if zebrafish metabolize and excrete drugs in the same fashion as mammals. Therefore, these issues may present a caveat when translating results into human clinical application.

**FIGURE 3:**
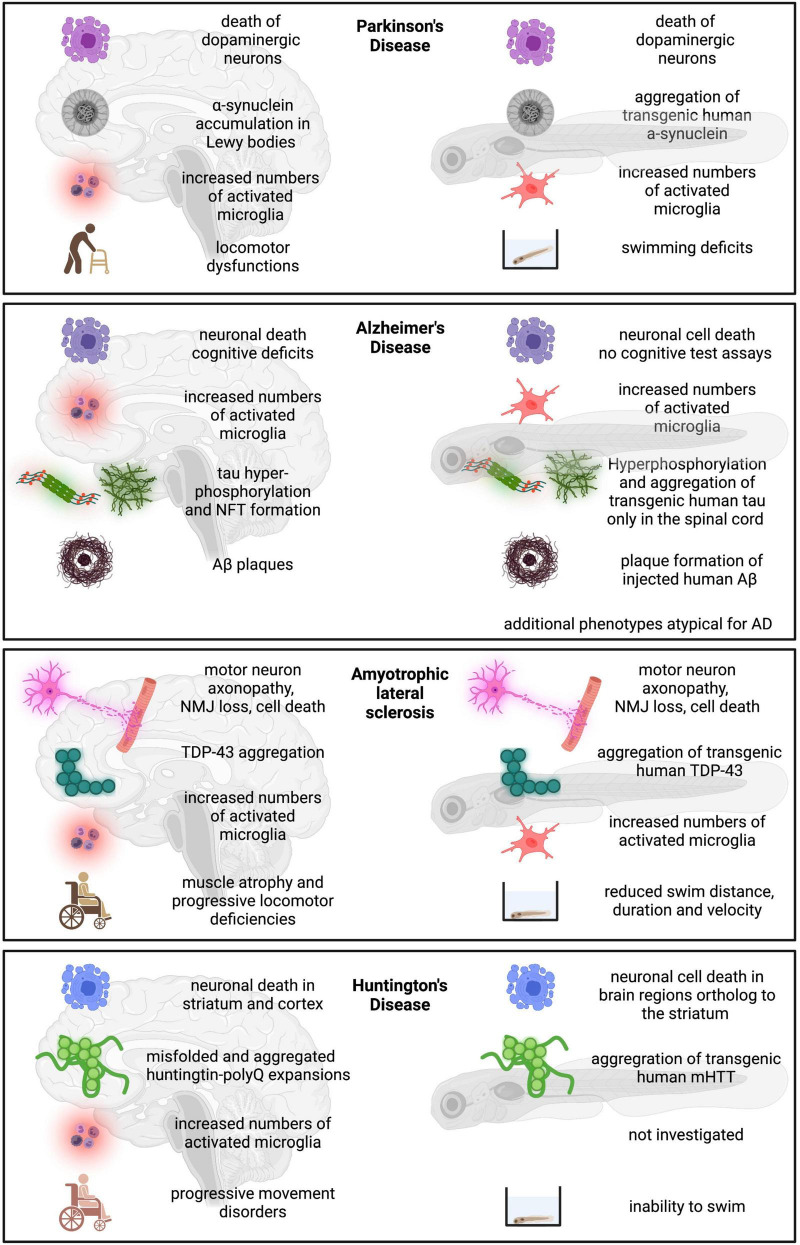
Schematic overview of zebrafish model compared to human disease condition. Human disease condition on the left compared to zebrafish phenotypes on the right.

Research into PD pathology has already integrated the zebrafish model well (Figure 3). Transgenic expression of human α-synuclein and knockdown of genes carrying mutations in PD patients (*PINK1*, *LRRK2*, *Parkin*, *DJ-1*, and *FBXO7*) have recapitulated the classic symptoms of the disease such as loss of dopaminergic neurons, neuroinflammation, locomotor and behavioral dysfunctions. Similarly, neurotoxin and pesticide exposure (MPTP, 6-OHDA, ziram, rotenone, and paraquat) resulted in loss of dopaminergic neurons as well as locomotor impairments. The zebrafish model thus presents a platform to investigate mechanisms of aggregate formation and consequences of gene mutation or pesticide exposure in the CNS. Several studies also included behavioral tests to measure depression, anxiety, social interaction aversion and aggression, features that are present in many PD patients. However, the results were quite variable and there is a need to establish better behavioral screens for the psychiatric symptoms of PD in the zebrafish model.

Many recent studies have employed zebrafish to model AD, and despite successful recapitulation of Aβ-sheet aggregation, increased neuronal toxicity and death, modeling AD in the zebrafish has thus far been limited (Figure 3). Deletions of the *PSEN* genes were viable and did not exhibit morphological or neurological defects. Tau phosphorylation and NFTs formation were successfully induced, but only appeared in the spinal cord and not in the brain of the larval zebrafish. Aβ aggregation could only be observed when human Aβ was injected into the fish brain, but not from endogenous protein. Additionally, Aβ overexpression induced phenotypes atypical of clinical AD, such as increased neuronal progenitor plasticity and proliferation, and enhanced neurogenesis. However, treatment of fish larvae with the known protein phosphatase inhibitor OKA successfully induced tau hyperphosphorylation, as well as deposition of Aβ, formation of senile plaques, and induced learning and memory deficits in zebrafish, mimicking the classical hallmarks of AD. With the number of AD cases expected to continually rise over the coming years, the need for drug discoveries is imperative. Thus, recent efforts have focused on the advantages of the zebrafish as a cost-effective and rapid *in vivo* pharmacological model for AD drug discovery. In this context, the use of CRISPR/Cas gene editing may help to establish better zebrafish models of AD.

While ALS research has been typically carried out in rodent models, data obtained in the zebrafish thus far is showing great promise for modeling ALS (Figure 3). Zebrafish mutants of *FUS*, *TDP-34*, *C9orf72*, and *SOD1* all displayed hallmarks of ALS such as muscle atrophy, motor axon abnormalities, motor neuron loss, as well as behavioral and motor deficits. Importantly, zebrafish studies have greatly advanced our understanding of the molecular mechanisms involved in ALS pathogenesis. For example, analysis of *tardbp* null fish revealed that the actin cross-linking protein Filamin C was upregulated as part of TDP-43 mediated pathogenesis, a fact later confirmed in the analysis of human samples. Investigation into FUS function in zebrafish revealed that FUS is essential for the interaction between U1 snRNP and RNA Polymerase II (RNAP II) during splicing, thus physically and functionally coupling transcription to splicing (250). BPA treatment of zebrafish not only induced motor axonopathy and loss of neuromuscular junction integrity, but was also associated with increased numbers of apoptotic motor neurons and the presence of activated microglia. Importantly, the zebrafish may prove to be particularly useful when disease phenotypes fail to manifest in classical mammalian models. For example, while knockdown of *C9orf72* in zebrafish led to typical symptoms of ALS, previous studies in rodent models failed to show adverse effects of *C9orf72* deficiency on neural health (Lagier-Tourenne et al., 2013; Burberry et al., 2016). Finally, the excellent accessibility for imaging and optogenetics allows for real time analysis of developing axon pathology and motor neuron death overtime in zebrafish.

In comparison to other disease models, not many studies have been carried out to explore HD pathology in the zebrafish. The overexpression of human mHTT, and different *Htt* knockdown analyses showed increased apoptosis and neuronal cell death in brain regions ortholog to those affected in HD patients (like the striatum), and disturbed neural tube formation. Importantly, zebrafish are viable in the absence of Huntingtin in contrast to mice, which has helped to better understand the neurodevelopmental aspects of HD. Studies in zebrafish revealed that impaired HTT function in early development could have limiting effects on precursor cell numbers of neurons affected by the loss of normal HTT in later development. Other studies identified REST as a molecular target of HTT in regulating BDNF expression and strikingly, found that the soluble mutant forms of HTT mRNA and protein were the toxic components causing cell death. Two anti-prion compounds were identified that successfully inhibited poly-Q aggregation. Finally, zebrafish transgenic for a truncated form of mHTT (mHTT-ΔN17) displayed a rapidly progressive movement disorder, reminiscent of that seen in other animal models and human patients (Figure 3).

To find new therapeutics for neurodegenerative diseases, the blood brain barrier constitutes a major hinderance for drugs to reach their targets in the brain. Nanotechnology is becoming a promising field of research for brain drug delivery using nanosized particles. Here the zebrafish is also proving an advantageous model for assessing blood brain barrier permeability with respect to novel neuro-specific technologies. Zebrafish provide an excellent model to test nanoparticle biocompatibility and toxicity (Chakraborty et al., 2016; White et al., 2019), and recent studies show success in treatment with nanoparticles to provide neuroprotection from Aβ induced toxicity as seen in AD (Saleem et al., 2022).

In summary, current data encourage the use of zebrafish as a model for neurodegenerative diseases. While we have described some of the limitations, the zebrafish presents a rapid, cost effective, and highly practical platform compared to classical mammalian models. Recent analyses employing cutting edge technology such as optogenetics have provided new insights into processes such as aggregate formation and the downstream consequences on a molecular level. Moreover, new tools such as very early cell death reporters make zebrafish an asset in neurodegeneration research. The plethora of cellular fluorescent reporters and cutting-edge imaging techniques, combined with the accessibility for drug testing, underscores the potential of zebrafish for the development of therapies for human neurodegenerative diseases.

## Author contributions

KC, AK, DS, and JP contributed to the conceptualization and writing – review and editing. DS and JP contributed to the resources, supervision, and project administration. KC and AK contributed to the writing – original draft and visualization. JP contributed to the funding acquisition. All authors contributed to the article and approved the submitted version.
